# Supplementary material for: Endophytic Diaporthe Associated with Morinda officinalis in China
Source: J Fungi (Basel). 2022 Jul 29;8(8):806. doi: 10.3390/jof8080806 (PMC9410054; doi:10.3390/jof8080806)
Supplement: Supplementary file 1 [file jof-08-00806-s001.zip › jof-1762643-supplementary.pdf]

Journal of Fungi

**Table S1** Collection details and GenBank accession numbers of isolates included in this study

| Species | Culture collection/ Herbarium number | GenBank accession numbers | | | | |
|---|---|---|---|---|---|---|
| | | ITS | *tub2* | *his* | *tef1-α* | *cal* |
| *D. acaciarum* | CBS 138862* | KP004460 | KP004509 | KP004504 | – | – |
| *D. acaciigena* | CBS 129521* | KC343005 | KC343973 | KC343489 | KC343731 | KC343247 |
| *D. acericola* | MFLUCC 17-0956* | KY964224 | KY964074 | – | KY964180 | KY964137 |
| *D. acerina* | CBS 137.27 | KC343006 | KC343974 | KC343490 | KC343732 | KC343248 |
| *D. acuta* | PSCG 047* | MK626957 | MK691225 | MK726161 | MK654802 | MK691124 |
| *D. acutispora* | CGMCC 3.18285* | KX986764 | KX999195 | KX999235 | KX999155 | KX999274 |
| *D. alleghaniensis* | CBS 495.72* | FJ889444 | KC843228 | KC343491 | GQ250298 | KC343249 |
| *D. alnea* | CBS 146.46* | KC343008 | KC343976 | KC343492 | KC343734 | KC343250 |
| *D. ambigua* | CBS 114015* | KC343010 | KC343978 | KC343494 | KC343736 | KC343252 |
| *D. ampelina* | CBS 114016* | AF230751 | JX275452 | – | GQ250351 | JX197443 |
| *D. amygdali* | CBS 126679* | KC343022 | KC343990 | KC343506 | KC343748 | KC343264 |
| *D. anacardii* | CBS 720.97* | KC343024 | KC343992 | KC343508 | KC343750 | KC343266 |
| *D. angelicae* | CBS 111592* | KC343026 | KC343994 | KC343511 | KC343752 | KC343268 |
| *D. anhuiensis* | CNUCC 201901 | MN219718 | MN227008 | MN224556 | MN224668 | MN224549 |
| *D. apiculatum* | LC 3418* | KP267896 | KP293476 | – | KP267970 | – |
| *D. aquatica* | IFRDCC 3051* | JQ797437 | – | – | – | – |
| *D. araucanorum* | RGM 2546 | MN509711 | MN509722 | – | MN509733 | MN974277 |
| *D. arctii* | CBS 136.25 | KC343031 | KC343999 | KC343515 | KC343757 | KC343273 |
| *D. arecae* | CBS 161.64* | KC343032 | KC344000 | KC343516 | KC343758 | KC343274 |
| *D. arengae* | CBS 114979* | KC343034 | KC344002 | KC343518 | KC343760 | KC343276 |
| *D. arezzoensis* | MFLU 19-2880* | MT185503 | MT454055 | – | MT454019 | – |
| *D. aseana* | MFLUCC 12-0299a* | KT459414 | KT459432 | – | KT459448 | KT459464 |
| *D. asheicola* | CBS 136967* | KJ160562 | KJ160518 | – | KJ160594 | KJ160542 |
| *D. albosinensis* | CFCC 53066* | MK432659 | MK578059 | MK443004 | MK578133 | MK442979 |
| *D. aspalathi* | CBS 117169* | KC343036 | KC344004 | KC343520 | KC343762 | KC343278 |
| *D. australafricana* | CBS 111886* | KC343038 | KC344006 | KC343522 | KC343764 | KC343280 |
| *D. australiana* | BRIP 66145* | MN708222 | MN696530 | – | MN696522 | – |
| | BRIP 66146 | MN708223 | MN696531 | – | – | – |
| | BRIP 66147 | MN708224 | MN696532 | – | MN696523 | – |
| *D. baccae* | CBS 136972* | KJ160565 | MF418509 | MF418264 | KJ160597 | – |
| *D. batatas* | CBS 122.21 | KC343040 | KC344008 | KC343524 | KC343766 | KC343282 |
| *D. bauhiniae* | CFCC 53071 | MK432648 | MK578051 | MK442995 | MK578124 | MK442970 |
| *D. beckhausii* | CBS 138.27 | KC343041 | KC344009 | KC343525 | KC343767 | KC343283 |
| *D. beilharziae* | BRIP 54792* | JX862529 | KF170921 | – | JX862535 | – |
| *D. benedicti* | CFCC 50062* | KP208847 | KP208855 | KP208851 | KP208853 | KP208849 |
| *D. betulae* | CFCC 50469* | KT732950 | KT733020 | KT732999 | KT733016 | KT732997 |
| *D. betulicola* | CFCC 51128* | KX024653 | KX024657 | KX024661 | KX024655 | KX024659 |
| *D. bicincta* | CBS 121004* | KC343134 | KC344102 | KC343618 | KC343860 | KC343376 |

| | | | | | | |
|---|---|---|---|---|---|---|
| *D. biconispora* | CGMCC 3.17252* | KJ490597 | KJ490418 | KJ490539 | KJ490476 | – |
| | ZJUD62 | KJ490597 | KJ490418 | KJ490539 | KJ490476 | MT898460 |
| *D. biguttulata* | ICMP20657* | KJ490582 | KJ490403 | KJ490524 | KJ490461 | – |
| | ZJUD48 | KJ490583 | KJ490404 | KJ490525 | KJ490462 | – |
| *D. biguttusis* | CGMCC 3.17081* | KF576282 | KF576306 | – | KF576257 | – |
| *D. bohemiae* | CBS 143347* | MG281015 | MG281188 | MG281361 | MG281536 | MG281710 |
| *D. brasiliensis* | CBS 133183* | KC343042 | KC344010 | KC343526 | KC343768 | KC343284 |
| *D. caatingaensis* | CBS 141542* | KY085927 | KY115600 | KY115605 | KY115603 | KY115597 |
| *D. camelliaesinensis* | SAUCC194.92* | MT822620 | MT855817 | MT855588 | MT855932 | MT855699 |
| *D. camptothecicola* | CFCC 51632* | KY203726 | KY228893 | KY228881 | KY228887 | KY228877 |
| *D. canthii* | CBS 132533* | JX069864 | KC843230 | – | KC843120 | KC843174 |
| *D. carpini* | CBS 114437 | KC343044 | KC344012 | KC343528 | KC343770 | KC343286 |
| *D. cassines* | CBS 136440* | KF777155 | – | – | KF777244 | – |
| *D. caulivora* | CBS 127268* | KC343045 | KC344013 | KC343529 | KC343771 | KC343287 |
| *D. celastrina* | CBS 139.27* | KC343047 | KC344015 | KC343531 | KC343773 | KC343289 |
| *D. celeris* | CBS 143349* | MG281017 | MG281190 | MG281363 | MG281538 | MG281712 |
| *D. ceratozamiae* | CBS 131306* | JQ044420 | – | – | – | – |
| *D. chamaeropis* | CBS 454.81 | KC343048 | KC344016 | KC343532 | KC343774 | KC343290 |
| *D. charlesworthii* | BRIP 54884m* | KJ197288 | KJ197268 | – | KJ197250 | – |
| *D. chongqingensis* | PSCG435* | MK626916 | MK691321 | MK726257 | MK654866 | MK691209 |
| ***D. chongqingensis*** | **ZHKUCC 22-0043** | **ON322872** | **ON315062** | **–** | **ON315034** | **–** |
| *D. chrysalidocarpi* | SAUCC194.35* | MT822563 | MT855760 | MT855532 | MT855876 | MT855646 |
| *D. cichorii* | MFLUCC 17-1023* | KY964220 | KY964104 | – | KY964176 | KY964133 |
| *D. cinerascens* | CBS 719.96 | KC343050 | KC344018 | KC343534 | KC343776 | KC343292 |
| *D. cissampeli* | CBS 141331* | KX228273 | KX228384 | KX228366 | – | – |
| *D. citri* | CBS 135422* | KC843311 | KC843187 | MF418281 | KC843071 | KC843157 |
| *D. citriasiana* | CBS 134240* | JQ954645 | KC357459 | MF418282 | JQ954663 | *KC357491* |
| *D. citrichinensis* | CBS 134242* | JQ954648 | MF418524 | KJ420880 | JQ954666 | KC357494 |
| *D. compacta* | LC3083* | KP267854 | KP293434 | KP293508 | KP267928 | – |
| *D. constrictospora* | CGMCC 3.20096 | MT385947 | MT424702 | MW022487 | MT424682 | MT424718 |
| *D. convolvuli* | CBS 124654 | KC343054 | KC344022 | KC343538 | KC343780 | KC343296 |
| *D. coryli* | CFCC 53083* | MK432661 | MK578061 | MK443006 | MK578135 | MK442981 |
| *D. crataegi* | CBS 114435 | KC343055 | KC344023 | KC343539 | KC343781 | KC343297 |
| *D. crotalariae* | CBS 162.33* | KC343056 | KC344024 | KC343540 | KC343782 | KC343298 |
| *D. crousii* | CAA823* | MK792311 | MK837932 | MK871450 | MK828081 | MK883835 |
| *D. cucurbitae* | DAOM 42078* | KM453210 | KP118848 | KM453212 | KM453211 | – |
| *D. cuppatea* | CBS 117499* | AY339322 | JX275420 | KC343541 | AY339354 | JX197414 |
| *D. cynaroidis* | CBS 122676 | KC343058 | KC344026 | KC343542 | KC343784 | KC343300 |

| | | | | | | |
|---|---|---|---|---|---|---|
| *D. cytosporella* | CBS 137020* | KC843307 | KC843221 | *MF418283* | KC843116 | KC843141 |
| *D. decedens* | CBS 109772 | KC343059 | KC344027 | KC343543 | KC343785 | KC343301 |
| *D. decorticans* | CBS 114200 | KC343169 | KC344137 | KC343653 | KC343895 | KC343411 |
| *D. destruens* | SPL15025* | MH465671 | – | – | MH560611 | MH560612 |
| *D. detrusa* | CBS 109770 | KC343061 | KC344029 | KC343545 | KC343787 | KC343303 |
| *D. diospyricola* | CBS 136552* | KF777156 | – | – | – | – |
| *D. discoidispora* | ICMP20662* | KJ490624 | KJ490445 | KJ490566 | KJ490503 | – |
| *D. dorycnii* | MFLUCC 17-1015* | KY964215 | KY964099 | – | KY964171 | – |
| *D. drenthii* | BRIP 66524* | MN708229 | MN696537 | – | MN696526 | – |
| *D. durionigena* | KCSR1812.8* | MN453530 | MT276159 | – | MT276157 | – |
| *D. elaeagni-glabrae* | CGMCC 3.18287* | KX986779 | KX999212 | KX999251 | KX999171 | KX999281 |
| *D. eleagni* | CBS 504.72 | KC343064 | KC344032 | KC343548 | KC343790 | KC343306 |
| *D. ellipicola* | CGMCC 3.17084* | KF576270 | KF576291 | – | KF576245 | – |
| *D. ellipsospora* | CGMCC 3.20099 | MT385949 | MT424704 | MW022488 | MT424684 | MT424720 |
| *D. endocitricola* | ZHKUCC 20-0012* | MT355682 | MT409290 | – | MT409336 | MT409312 |
| *D. endophytica* | CBS 133811* | KC343065 | KC344033 | KC343549 | KC343791 | KC343307 |
| *D. eres* | CBS 138594* | KJ210529 | KJ420799 | KJ420850 | KJ210550 | KJ434999 |
| *D. eucalyptorum* | CBS 132525* | JX069862 | – | – | – | – |
| ***D. eucalyptorum*** | **ZHKUCC 22-0044** | **ON322873** | **ON315063** | **–** | **ON315035** | **–** |
| ***D. eucalyptorum*** | **ZHKUCC 22-0045** | **ON322874** | **ON315064** | **ON315006** | **ON315036** | **–** |
| *D. eugeniae* | CBS 444.82 | KC343098 | KC344066 | KC343582 | KC343824 | KC343340 |
| *D. fibrosa* | CBS 109751 | KC343099 | KC344067 | KC343583 | KC343825 | KC343341 |
| *D. foeniculacea* | CBS 123208 | KC343104 | KC344072 | KC343588 | KC343830 | KC343346 |
| *D. foeniculina* | CBS 111553* | KC343101 | KC344069 | KC343585 | KC343827 | KC343343 |
| *D. foeniculina* | MFLUCC 20-0151 | MW020272 | MW057340 | MW057341 | – | – |
| *D. foeniculina* | MFLUCC 17-1068 | KY964188 | KY964071 | – | KY964144 | – |
| *D. foeniculina* | MFLUCC 17-1020 | KY964218 | KY964102 | – | KY964174 | – |
| *D. foeniculina* | AR5145 | KC843306 | KC843220 | – | KC843115 | KC843140 |
| *D. foeniculina* | DP0454 | KC843297 | KC843211 | – | KC843106 | KC843131 |
| *D. foikelawen* | RGM 2539* | MN509713 | MN509724 | – | MN509735 | MN974278 |
| *D. foikelawen* | CHL: RGM 2539 | MN509713 | MN509724 | | MN509735 | MN974278 |
| *D. fraxini-angustifoliae* | BRIP 54781* | JX862528 | KF170920 | – | JX852534 | – |
| *D. fructicola* | MAFF 246408* | LC342734 | LC342736 | LC342737 | LC342735 | LC342738 |
| *D. fulvicolor* | PSCG 051* | MK626859 | MK691236 | MK726163 | MK654806 | MK691132 |
| *D. fusicola* | CGMCC 3.17087* | KF576281 | KF576305 | – | KF576256 | KF576233 |
| *D. ganjae* | CBS 180.91* | KC343112 | KC344080 | KC343596 | KC343838 | KC343354 |
| *D. ganzhouensis* | CFCC 53087 | MK432665 | MK578065 | MK443010 | MK578139 | MK442985 |

| | | | | | | |
|---|---|---|---|---|---|---|
| *D. gardeniae* | CBS 288.56 | KC343113 | KC344081 | KC343597 | KC343839 | KC343355 |
| *D. garethjonesii* | MFLUCC 12-0542a* | KT459423 | KT459441 | – | KT459457 | KT459470 |
| *D. goulteri* | BRIP 55657a* | KJ197290 | KJ197270 | – | KJ197252 | – |
| *D. grandiflori* | SAUCC194.84* | MT822612 | MT855809 | MT855580 | MT855924 | MT855691 |
| *D. guangdongensis* | ZHKUCC 20-0014* | MT355684 | MT409292 | – | MT409338 | MT409314 |
| *D. guangxiensis* | JZB320094* | MK335772 | MK500168 | – | MK523566 | MK736727 |
| ***D. guangxiensis*** | **ZHKUCC 22-0046** | **ON322875** | **ON315065** | **ON315007** | **ON315037** | **ON314994** |
| *D. gulyae* | BRIP 54025* | JF431299 | KJ197271 | – | JN645803 | – |
| *D. guttulata* | CGMCC 3.20100 | MT385950 | MT424705 | MW022491 | MT424685 | MW022470 |
| *D. helianthi* | CBS 592.81* | KC343115 | KC344083 | KC343599 | KC343841 | JX197454 |
| *D. heliconiae* | SAUCC194.77* | MT822605 | MT855802 | MT855573 | MT855917 | MT855684 |
| ***D. heliconiae*** | **ZHKUCC 22-0047** | **ON322876** | **ON315066** | **ON315008** | **ON315038** | – |
| ***D. heliconiae*** | **ZHKUCC 22-0048** | **ON322877** | **ON315067** | **ON315009** | – | – |
| *D. heterophyllae* | CBS 143769* | MG600222 | MG600226 | MG600220 | MG600224 | MG600218 |
| *D. heterostemmatis* | SAUCC194.85* | MT822613 | MT855810 | MT855581 | MT855925 | MT855692 |
| *D. heveae* | CBS 681.84 | KC343117 | KC344085 | KC343601 | KC343843 | KC343359 |
| *D. heveae* | CBS 852.97 | KC343116 | KC344084 | KC343600 | KC343842 | KC343358 |
| *D. hickoriae* | CBS 145.26* | KC343118 | KC344086 | KC343602 | KC343844 | KC343360 |
| *D. hispaniae* | CBS 143351* | MG281123 | MG281296 | MG281471 | MG281644 | MG281820 |
| *D. hongkongensis* | CBS 115448* | KC343119 | KC344087 | KC343603 | KC343845 | KC343361 |
| *D. hordei* | CBS 481.92 | KC343120 | KC344088 | KC343604 | KC343846 | KC343362 |
| *D. huangshanensis* | CNUCC 201903* | MN219729 | MN227010 | MN224558 | MN224670 | – |
| *D. hubeiensis* | JZB320123* | MK335809 | MK500148 | – | MK523570 | MK500235 |
| | JZB320122 | MK335808 | MK500146 | – | MK523569 | MK500234 |
| *D. hungariae* | CBS 143353* | MG281126 | MG281299 | MG281474 | MG281647 | MG281823 |
| *D. impulsa* | CBS 114434 | KC343121 | KC344089 | KC343605 | KC343847 | KC343363 |
| *D. incompleta* | CGMCC 3.18288* | KX986794 | KX999226 | KX999265 | KX999186 | KX999289 |
| *D. inconspicua* | CBS 133813* | KC343123 | KC344091 | KC343607 | KC343849 | KC343365 |
| *D. infecunda* | CBS 133812* | KC343126 | KC344094 | KC343610 | KC343852 | KC343368 |
| *D. irregularis* | CGMCC 3.20092 | MT385951 | MT424706 | – | MT424686 | MT424721 |
| *D. isoberliniae* | CBS 137981* | KJ869133 | KJ869245 | – | – | – |
| *D. italiana* | MFLUCC:18-0091* | MH846238 | MH853689 | – | MH853687 | MH853691 |
| *D. juglandicola* | CFCC 51134* | KU985101 | KX024634 | – | KX024628 | KX024616 |
| *D. kochmanii* | BRIP 54033* | JF431295 | – | – | JN645809 | – |
| *D. kongii* | BRIP 54031* | JF431301 | KJ197272 | – | JN645797 | – |
| *D. krabiensis* | MFLUCC 17-2481* | MN047100 | MN431495 | – | MN433215 | – |
| *D. lenispora* | CGMCC 3.20101 | MT385952 | MT424707 | MW022493 | MT424687 | MW022472 |
| *D. leucospermi* | CBS 111980* | JN712460 | KY435673 | KY435653 | KY435632 | KY435663 |
| *D. limonicola* | CBS 142549* | MF418422 | MF418582 | MF418342 | MF418501 | MF418256 |

| | | | | | | |
|---|---|---|---|---|---|---|
| *D. litchicola* | BRIP 54900* | JX862533 | KF170925 | – | JX862539 | – |
| *D. litchii* | AUCC194.22* | MT822550 | MT855747 | MT855519 | MT855863 | MT855635 |
| *D. lithocarpus* | CGMCC 3.15175* | KC153104 | KF576311 | – | KC153095 | – |
| *D. longicicola* | CGMCC 3.17089* | KF576267 | KF576291 | – | KF576242 | – |
| *D. longicolla* | FAU 599* | KJ590728 | KJ610883 | KJ659188 | KJ590767 | KJ612124 |
| | FAU 644 | KJ590730 | KJ610885 | KJ659190 | KJ590769 | KJ612126 |
| | FAU 657 | KJ590727 | KJ610882 | KJ659187 | KJ590766 | KJ612123 |
| *D. longispora* | CBS 194.36* | KC343135 | KC344103 | KC343619 | KC343861 | KC343377 |
| ***D. longticola*** | **ZHKUCC 22-0058** | **ON322887** | **ON315076** | **ON315017** | **ON315044** | – |
| ***D. longticola*** | **ZHKUCC 22-0059** | **ON322888** | **ON315077** | **ON315018** | – | – |
| ***D. longticola*** | **ZHKUCC 22-0060** | **ON322889** | **ON315078** | **ON315019** | **ON315045** | – |
| ***D. longticola*** | **ZHKUCC 22-0061** | **ON322890** | **ON315079** | **ON315020** | **ON315046** | – |
| ***D. longticola*** | **ZHKUCC 22-0062** | **ON322891** | **ON315080** | **ON315021** | **ON315047** | – |
| ***D. longticola*** | **ZHKUCC 22-0063** | **ON322892** | **ON315081** | **ON315022** | **ON315048** | – |
| ***D. longticola*** | **ZHKUCC 22-0064** | **ON322893** | **ON315082** | **ON315023** | **ON315049** | – |
| ***D. longticola*** | **ZHKUCC 22-0065** | **ON322894** | **ON315083** | **ON315024** | **ON315050** | – |
| ***D. longticola*** | **ZHKUCC 22-0066** | – | **ON315084** | – | **ON315051** | **–** |
| *D. lonicerae* | MFLUCC 17-0963* | KY964190 | KY964073 | – | KY964146 | KY964116 |
| *D. lusitanicae* | CBS 123212* | KC343136 | KC344104 | KC343620 | KC343862 | KC343378 |
| *D. lutescens* | SAUCC194.36* | MT822564 | MT855761 | MT855533 | MT855877 | MT855647 |
| *D. macadamiae* | BRIP 66526* | MN708230 | MN696539 | – | MN696528 | – |
| *D. machili* | SAUCC194.111* | MT822639 | MT855836 | MT855606 | MT855951 | MT855718 |
| *D. macintoshii* | BRIP 55064a* | KJ197289 | KJ197269 | – | KJ197251 | – |
| *D. mahothocarpus* | CGMCC 3.15181 | KC153096 | – | – | KC153087 | – |
| *D. malorum* | CBS142383* | KY435638 | KY435668 | KY435648 | KY435627 | KY435658 |
| *D. manihotia* | CBS 505.76 | KC343138 | KC344106 | KC343622 | KC343864 | KC343380 |
| *D. marina* | MFLU 17-2622* | MN047102 | – | – | – | – |
| *D. maritima* | DAOMC 250563* | KU552025 | KU574615 | – | KU552023 | – |
| *D. masirevicii* | BRIP 57892a* | KJ197277 | KJ197257 | – | KJ197239 | – |
| *D. mayteni* | CBS 133185* | KC343139 | KC344107 | KC343623 | KC343865 | KC343381 |
| *D. maytenicola* | CBS 136441* | KF777157 | KF777250 | – | – | – |
| *D. megalospora* | CBS 143.27 | KC343140 | KC344108 | KC343624 | KC343866 | KC343382 |
| ***D. megabispora*** | **ZHKUCC 22-0067** | **ON322895** | **ON315085** | **ON315025** | – | **ON315001** |
| ***D. megabispora*** | **ZHKUCC 22-0068** | **ON322896** | **ON315086** | **ON315026** | **ON315052** | – |
| *D. melastomatis* | SAUCC194.55* | MT822583 | MT855780 | MT855551 | MT855896 | MT855664 |
| *D. melitensis* | CBS 142551* | MF418424 | MF418584 | MF418344 | MF418503 | MF418258 |
| *D. melonis* | CBS 507.78* | KC343142 | KC344110 | KC343626 | KC343868 | KC343384 |
| *D. middletonii* | BRIP 54884e* | KJ197286 | KJ197266 | – | KJ197248 | – |
| *D. millettiae* | GUCC9167* | MK398674 | MK460488 | – | MK480609 | MK502086 |
| *D. minima* | CGMCC 3.20097 | MT385953 | MT424708 | MW022496 | MT424688 | MT424722 |
| *D. minusculata* | CGMCC 3.20098 | MT385957 | MT424712 | MW022499 | MT424692 | MW022475 |
| *D. miriciae* | BRIP 54736j* | KJ197283 | KJ197263 | – | KJ197245 | – |

| | | | | | | |
|---|---|---|---|---|---|---|
| *D. momicola* | MFLUCC 16-0113* | KU557563 | KU557587 | – | KU557631 | KU557611 |
| ***D. morinendophytica*** | **ZHKUCC 22-0069** | **ON322897** | **ON315087** | **ON315027** | **ON315053** | – |
| ***D. morinendophytica*** | **ZHKUCC 22-0070** | **ON322898** | **ON315088** | **ON315028** | **ON315054** | – |
| ***D. morinendophytica*** | **ZHKUCC 22-0071** | **ON322899** | **ON315089** | **ON315029** | **ON315055** | **ON315002** |
| ***D. morindae*** | **ZHKUCC 22-0072** | **ON322100** | **ON315090** | **ON315030** | **ON315056** | **ON315003** |
| ***D. morindae*** | **ZHKUCC 22-0073** | **ON322101** | **ON315091** | **ON315031** | **ON315057** | **ON315004** |
| ***D. morindae*** | **ZHKUCC 22-0074** | **ON322102** | **ON315092** | **ON315032** | **ON315058** | **ON315005** |
| ***D. morindae*** | **ZHKUCC 22-0075** | **ON322103** | **ON315093** | **ON315033** | **ON315059** | – |
| ***D. morindae*** | **ZHKUCC 22-0076** | **ON322104** | **ON315094** | – | **ON315060** | – |
| *D. multigutullata* | ICMP20656* | KJ490633 | KJ490454 | KJ490575 | KJ490512 | – |
| *D. musigena* | CBS 129519* | KC343143 | KC344111 | KC343627 | KC343869 | KC343385 |
| *D. myracrodruonis* | URM 7972* | NR_163320 | MK205291 | – | MK213408 | MK205290 |
| *D. neilliae* | CBS 144.27* | KC343144 | KC344112 | KC343628 | KC343870 | KC343386 |
| *D. neoarctii* | CBS 109490 | KC343145 | KC344113 | KC343629 | KC343871 | KC343387 |
| *D. nomurai* | CBS 157.29 | KC343154 | KC344122 | KC343638 | KC343880 | KC343396 |
| *D. nothofagi* | BRIP 54801* | JX862530 | KF170922 | – | JX862536 | – |
| *D. novem* | CBS 127271* | KC343157 | KC344125 | KC343641 | KC343883 | KC343399 |
| *D. obtusifoliae* | CBS 143449* | MG386072 | – | MG386137 | – | – |
| *D. ocoteae* | CBS 141330* | KX228293 | KX228388 | – | – | – |
| *D. oncostoma* | CBS 589.78 | KC343162 | KC344130 | KC343646 | KC343888 | KC343404 |
| *D. oraccinii* | LC 3166* | KP267863 | KP293443 | KP293517 | KP267937 | – |
| *D. osmanthi* | GUCC9165* | MK398675 | MK502091 | – | MK480610 | MK502087 |
| *D. ovalispora* | ICMP20659* | KJ490628 | KJ490449 | KJ490570 | KJ490507 | – |
| *D. ovoicicola* | CGMCC 3.17092* | KF576264 | KF576288 | – | KF576239 | KF576222 |
| *D. oxe* | CBS 133186* | KC343164 | KC344132 | KC343648 | KC343890 | KC343406 |
| *D. paranensis* | CBS 133184 | KC343171 | KC344139 | KC343655 | KC343897 | KC343413 |
| *D. parapterocarpi* | CBS 137986* | KJ869138 | KJ869248 | – | – | – |
| *D. parvae* | PSCG 034* | MK626919 | MK691248 | MK726210 | MK654858 | – |
| *D. pascoei* | BRIP 54847* | JX862532 | KF170924 | – | JX862538 | – |
| *D. passiflorae* | CBS 132527* | JX069860 | KY435674 | KY435654 | KY435633 | KY435664 |
| *D. passifloricola* | CBS 141329* | KX228292 | KX228387 | KX228367 | – | – |
| *D. patagonica* | RGM 2473* | MN509717 | MN509728 | – | MN509739 | MN974279 |
| *D. patagonica* | CHL: RGM 2473 | MN509717 | MN509728 | | MN509739 | MN974279 |
| *D. penetriteum* | LC 3353 | KP714505 | KP714529 | KP714493 | KP714517 | – |
| *D. perjuncta* | CBS 109745* | KC343172 | KC344140 | KC343656 | KC343898 | KC343414 |

| | | | | | | |
|---|---|---|---|---|---|---|
| *D. perniciosa* | CBS 124030 | KC343149 | KC344117 | KC343633 | KC343875 | KC343391 |
| *D. perseae* | CBS 151.73 | KC343173 | KC344141 | KC343657 | KC343899 | KC343415 |
| *D. pescicola* | MFLUCC 16-0105* | KU557555 | KU557579 | – | KU557623 | KU557603 |
| *D. phaseolorum* | CBS 113425 | KC343174 | KC344142 | KC343658 | KC343900 | KC343416 |
| *D. phillipsii* | MUM 19.28* | MK792305 | MN000351 | MK871445 | MK828076 | MK883831 |
| *D. phragmitis* | CBS 138897* | KP004445 | KP004507 | KP004503 | – | – |
| *D. podocarpi-macrophylli* | CGMCC3.18281* | KX986774 | KX999207 | KX999246 | KX999167 | KX999278 |
| *D. pometiae* | SAUCC194.72* | MT822600 | MT855797 | MT855568 | MT855912 | MT855679 |
| *D. pseudomangiferae* | CBS 101339* | KC343181 | KC344149 | KC343665 | KC343907 | KC343423 |
| *D. pseudophoenicicola* | CBS 462.69* | KC343184 | KC344152 | KC343668 | KC343910 | KC343426 |
| *D. pseudotsugae* | MFLU 15-3228 | KY964225 | KY964108 | – | KY964181 | KY964138 |
| *D. psoraleae* | CBS 136412* | KF777158 | KF777251 | – | KF777245 | – |
| *D. psoraleae-pinnatae* | CBS 136413* | KF777159 | KF777252 | – | – | – |
| *D. pterocarpi* | MFLUCC 10-0571 | JQ619899 | JX275460 | – | JX275416 | JX197451 |
| *D. pterocarpicola* | MFLUCC 10-0580a | JQ619887 | JX275441 | – | JX275403 | JX197433 |
| *D. pungensis* | SAUCC194.112* | MT822640 | MT855837 | MT855607 | MT855952 | MT855719 |
| *D. pustulata* | CBS 109742 | KC343185 | KC344153 | KC343669 | KC343911 | KC343427 |
| *D. pyracanthae* | CBS142384* | KY435635 | KY435666 | KY435645 | KY435625 | KY435656 |
| *D. racemosae* | CBS 143770* | MG600223 | MG600227 | MG600221 | MG600225 | MG600219 |
| *D. raonikayaporum* | CBS 133182* | KC343188 | KC344156 | KC343672 | KC343914 | KC343430 |
| *D. ravennica* | MFLUCC 15-0479* | KU900335 | KX432254 | – | – | – |
| *D. rhoina* | CBS 146.27 | KC343189 | KC344157 | KC343673 | KC343915 | KC343431 |
| *D. rhusicola* | MFLU 17-0647 | MG828893 | MG922552 | – | MG922551 | – |
| *D. rhusicola* | MFLUCC 16-1393 | KY684947 | KY684945 | – | KY684946 | – |
| *D. rosiphthora* | COAD 2914 | MT311197 | – | – | MT313693 | MT313691 |
| *D. rosiphthora* | COAD 2914* | MT311197 | – | – | – | – |
| *D. rossmaniae* | MUM 19.30* | MK792290 | MK837914 | MK871432 | MK828063 | MK883822 |
| *D. rostrata* | CFCC 50062* | KP208847 | KP208855 | KP208851 | KP208853 | KP208849 |
| *D. rudis* | CBS 113201 | KC343234 | KC344202 | KC343718 | KC343960 | KC343476 |
| *D. saccarata* | CBS 116311* | KC343190 | KC344158 | KC343674 | KC343916 | KC343432 |
| *D. sackstonii* | BRIP 54669b* | KJ197287 | KJ197267 | – | KJ197249 | – |
| *D. salicicola* | BRIP 54825* | JX862531 | KF170923 | – | JX862537 | – |
| *D. salinicola* | MFLUCC 18-0553 * | MN047098 | – | – | MN077073 | – |
| *D. sambucusii* | CFCC 51986* | KY852495 | KY852511 | KY852503 | KY852507 | KY852499 |

| | | | | | | |
|---|---|---|---|---|---|---|
| *D. schimae* | CFCC 53103 | MK432640 | MK578043 | MK442987 | MK578116 | MK442962 |
| *D. schini* | CBS 133181* | KC343191 | KC344159 | KC343675 | KC343917 | KC343433 |
| *D. schisandrae* | CFCC 51988* | KY852497 | KY852513 | KY852505 | KY852509 | KY852501 |
| *D. schoeni* | MFLU 15-1279* | KY964226 | KY964109 | – | KY964182 | KY964139 |
| *D. sclerotioides* | CBS 296.67* | KC343193 | KC344161 | KC343677 | KC343919 | KC343435 |
| *D. scobina* | CBS 251.38 | KC343195 | KC344163 | KC343679 | KC343921 | KC343437 |
| *D. searlei* | BRIP 66528* | MN708231 | MN696540 | – | – | – |
| *D. sennae* | CFCC 51636* | KY203724 | KY228891 | – | KY228885 | KY228875 |
| *D. sennicola* | CFCC 51634* | KY203722 | KY228889 | – | KY228883 | KY228873 |
| *D. serafiniae* | BRIP 55665a* | KJ197274 | KJ197254 | – | KJ197236 | – |
| *D. shaanxiensis* | CFCC 53106* | MK432654 | – | MK443001 | MK578130 | MK442976 |
| *D. shennongjiaensis* | CNUCC 201905* | MN216229 | MN227012 | MN224560 | MN224672 | MN224551 |
| *D. siamensis* | MFLUCC 10-0573a | JQ619879 | JX275429 | – | JX275393 | – |
| | MFLUCC 17_0591 | | | | | |
| ***D. siamensis*** | **ZHKUCC 22-0049** | **ON322878** | – | – | – | – |
| ***D. siamensis*** | **ZHKUCC 22-0050** | **ON322879** | **ON315068** | **ON315010** | – | **ON314995** |
| *D. sojae* | CBS 139282* | KJ590719 | KJ610875 | KJ659208 | KJ590762 | KJ612116 |
| *D. spinosa* | PSCG383* | MK626849 | MK691234 | MK726156 | MK654811 | MK691129 |
| *D. sterilis* | CBS 136969* | KJ160579 | KJ160528 | MF418350 | KJ160611 | KJ160548 |
| *D. stewartii* | CBS 193.36 | FJ889448 | – | – | GQ250324 | – |
| *D. stictica* | CBS 370.54 | KC343212 | KC344180 | KC343696 | KC343938 | KC343454 |
| *D. subclavata* | ICMP20663* | KJ490630 | KJ490451 | KJ490572 | KJ490509 | – |
| *D. subordinaria* | CBS 101711 | KC343213 | KC344181 | KC343697 | KC343939 | KC343455 |
| *D. taoicola* | MFLUCC 16-0117* | KU557567 | KU557591 | – | KU557635 | – |
| *D. tarchonanthi* | CPC 37479* | MT223794 | MT223733 | MT223759 | – | – |
| *D. tecomae* | CBS 100547 | KC343215 | KC344183 | KC343699 | KC343941 | KC343457 |
| *D. tectonae* | MFLUCC 12-0777* | KU712430 | KU743977 | – | KU749359 | KU749345 |
| | MFLUCC 14-1139 | KU712438 | KU743985 | – | KU749366 | KU749353 |
| | MFLUCC 14-1138 | KU712437 | KU743984 | – | KU749365 | KU749352 |
| *D. tectonendophytica* | MFLUCC 13-0471* | KU712439 | KU743986 | – | KU749367 | KU749354 |
| *D. tectonigena* | MFLUCC 12-0767* | KU712429 | KU743976 | – | KU749371 | KU749358 |
| *D. terebinthifolii* | CBS 133180* | KC343216 | KC344184 | KC343700 | KC343942 | KC343458 |
| *D. ternstroemia* | CGMCC 3.15183* | KC153098 | – | – | KC153089 | – |
| *D. thunbergii* | MFLUCC 10-0756a | JQ619893 | JX275449 | – | JX275409 | JX197440 |
| *D. toxicodendri* | FFPRI420987 | LC275192 | LC275224 | LC275216 | LC275216 | LC275200 |
| *D. tulliensis* | BRIP 62248a | KR936130 | KR936132 | – | KR936133 | – |
| *D. ueckerae* | FAU 656 | KJ590726 | KJ610881 | KJ659215 | KJ590747 | KJ612122 |
| *D. undulata* | CGMCC 3.18293* | KX986798 | KX999230 | KX999269 | KX999190 | – |
| *D. unshiuensis* | CGMCC3.17569* | KJ490587 | KJ490408 | KJ490529 | KJ490466 | – |
| | ZJUD49 | KJ490584 | KJ490405 | KJ490526 | KJ490463 | – |

| | | | | | | |
|---|---|---|---|---|---|---|
| | ZJUD50 | KJ490585 | KJ490406 | KJ490527 | KJ490464 | – |
| ***D. unshiuensis*** | **ZHKUCC 22-0051** | **ON322880** | **ON315069** | **ON315011** | **ON315039** | **ON314996** |
| ***D. unshiuensis*** | **ZHKUCC 22-0052** | **ON322881** | **ON315070** | **–** | **ON315040** | **–** |
| ***D. unshiuensis*** | **ZHKUCC 22-0053** | **ON322882** | **ON315071** | **ON315012** | **ON315041** | **ON314997** |
| *D. vaccinii* | CBS 160.32* | AF317578 | KC344196 | KC343712 | GQ250326 | KC343470 |
| *D. vacuae* | MUM 19.31* | MK792309 | MK837931 | MK871449 | MK828080 | MK883834 |
| *D. vangueriae* | CBS 137985* | KJ869137 | KJ869247 | – | – | – |
| *D. vawdreyi* | BRIP 57887a | KR936126 | KR936128 | – | KR936129 | – |
| *D. velutina* | CGMCC 3.18286* | KX986790 | KX999223 | KX999261 | KX999182 | – |
| *D. verniciicola* | CFCC 53109 | MK573944 | MK574639 | MK574599 | MK574619 | MK574583 |
| *D. vexans* | CBS 127.14 | KC343229 | KC344197 | KC343713 | KC343955 | KC343471 |
| *D. viniferae* | JZB320071* | MK341550 | MK500112 | – | MK500107 | MK500119 |
| *D. virgiliae* | CBS 138788* | KP247573 | KP247582 | – | – | – |
| *D. woodii* | CBS 558.93 | KC343244 | KC344212 | KC343728 | KC343970 | KC343486 |
| *D. woolworthii* | CBS 148.27 | KC343245 | KC344213 | KC343729 | KC343971 | KC343487 |
| *D. xishuangbanica* | CGMCC 3.18282* | KX986783 | KX999216 | KX999255 | KX999175 | – |
| | LC9707 | KX986783 | KX999216 | KX999255 | KX999175 | – |
| ***D. xishuangbanica*** | **ZHKUCC 22-0054** | **ON322883** | **ON315072** | **ON315013** | **ON315042** | **ON314998** |
| ***D. xishuangbanica*** | **ZHKUCC 22-0055** | **ON322884** | **ON315073** | **ON315014** | **–** | **ON314999** |
| *D. xunwuensis* | CFCC 53085 | MK432663 | MK578063 | MK443008 | MK578137 | MK442983 |
| *D. yunnanensis* | CGMCC 3.18289* | KX986796 | KX999228 | KX999267 | KX999188 | KX999290 |
| *D. zaobaisu* | PSCG031* | MK626922 | MK691245 | MK726207 | MK654855 | – |
| ***D. zhaoqingense*** | **ZHKUCC 22-0056** | **ON322885** | **ON315074** | **ON315015** | **–** | **ON315000** |
| ***D. zhaoqingense*** | **ZHKUCC 22-0057** | **ON322886** | **ON315075** | **ON315016** | **ON315043** | **–** |
| *Diaporthella corylina* | CBS 121124* | KC343004 | KC343972 | – | KC343730 | KC343246 |

AR, Collection of Systematic Mycology and Microbiology Laboratory; ATCC, American type culture collection; CBS, Centraalbu reau voor Schimmelcultures (Netherlands); ZJUD, Diaporthe strains collected in Zhejiang University (China); BRIP, Plant Pathology Herbarium, Dutton Park, Queensland (Australia); CGMCC, China General Microbial Culture Collection Center (China); MFLUCC, Mae Fah Luang University Culture Collection (Thailand); JZB, Culture collection of Institute of Plant and Environment Protection, Beijing Academy of Agriculture and Forestry Sciences (China); ZHKUCC, University of Agriculture and Engineering Culture Col lection(China). Sequences produced in this study are shown in bold. * ex-type or ex-epitype culture. ITS, internal transcribed spacers 1 and 2 together with 5.8S nrDNA; tub2, partial beta-tubulin gene; cal, partial calmodulin gene; his, histone H3 and EF-1$\alpha$, partial translation elongation factor 1-$\alpha$ gene.
